# Human chorion-derived mesenchymal stem cells suppress JAK2/STAT3 signaling and induce apoptosis of cholangiocarcinoma cell lines

**DOI:** 10.1038/s41598-022-15298-0

**Published:** 2022-07-05

**Authors:** Tanachapa Jantalika, Sirikul Manochantr, Pakpoom Kheolamai, Duangrat Tantikanlayaporn, Weerachai Saijuntha, Somchai Pinlaor, Arthit Chairoungdua, Luminita Paraoan, Chairat Tantrawatpan

**Affiliations:** 1 Division of Cell Biology, Department of Preclinical Sciences, Faculty of Medicine, Thammasat University, Pathumthani, 12120 Thailand; 2 Center of Excellence in Stem Cell Research, Thammasat University, Pathumthani, 12120 Thailand; 3 Biodiversity and Conservation Research Unit, Walai Rukhavej Botanical Research Institute (WRBRI), Mahasarakham University, Maha Sarakham, 44150 Thailand; 4 Department of Parasitology, Faculty of Medicine, Khon Kaen University, Khon Kaen, 40002 Thailand; 5 Department of Physiology, Faculty of Science, Mahidol University, Bangkok, 10400 Thailand; 6 Department of Biology, Faculty of Arts and Sciences, Edge Hill University, BioSciences Building, St Helens Road, Ormskirk, L39 4QP UK

**Keywords:** Cancer, Cell biology, Stem cells

## Abstract

Cholangiocarcinoma (CCA) is an aggressive malignancy arising from the damaged epithelial cells of the biliary tract. Previous studies have reported that the multi-potent mesenchymal stem cells (MSCs) activate a series of tumor signaling pathways by releasing several cytokines to influence tumor cell development. However, the roles and mechanisms of human chorion-derived MSCs (CH-MSCs) in cholangiocarcinoma progression have not been fully addressed. This present study aims to examine the effects of conditioned media derived from CH-MSCs (CH-CM) on CCA cell lines and investigate the respective underlying mechanism of action. For this purpose, MSCs were isolated from chorion tissue, and three cholangiocarcinoma cell lines, namely KKU100, KKU213A, and KKU213B, were used. MTT assay, annexin V/PI analysis, and JC-1 staining were used to assess the effects of CH-CM on proliferation and apoptosis of CCA cells, respectively. Moreover, the effect of CH-CM on caspase-dependent apoptotic pathways was also evaluated. The western blotting assay was also used for measuring the expression of JAK2/STAT3 signaling pathway-associated proteins. The results showed that CH-CM suppressed proliferation and promoted apoptosis of CCA cell lines. CH-CM treatment-induced loss of mitochondrial membrane potential (∆Ψm) in CCA cell lines. The factors presented in the CH-CM also inhibited JAK2/STAT3 signaling, reduced the expression of BCL-2, and increased BAX expression in CCA cells. In conclusion, our study suggests that the CH-CM has a potent anti-cancer effect on cholangiocarcinoma cells and thus provides opportunities for use in alternative cell therapy or in combination with a conventional chemotherapeutic drug to increase the efficiency of CCA treatment.

## Introduction

Cholangiocarcinoma (CCA) is an aggressive malignancy arising from the damaged epithelial cells of varying locations within the biliary tract and may arise anywhere along the intrahepatic or extrahepatic biliary tree^1^. The incidence of cholangiocarcinoma is increasing worldwide, and it is the second-highest cause of global cancer mortality^2^. Surgery is the main treatment option for CCA. However, options for resection are limited because this therapy cannot be used to treat widespread cancer. The vast majority of patients (∼ 70%) are diagnosed late due to a lack of specific symptoms^3^ and thus present advanced metastatic stages, with only a few (∼ 25%) being eligible for surgery^4^. Therefore, it is critical that new and effective therapies are developed to better control cancer proliferation and progression.

The signal transducer and activator of transcription 3 (STAT3) has emerged as a potential CCA therapeutic target because this signaling axis plays a key role in the inflammation associated with CCA carcinogenesis and the development of CCA^5^. STAT3 is an important member of the STAT family of transcription factors and a critical molecule of the JAK/STAT signaling pathway involved in the regulation of gene expression related to cell proliferation, survival, and progression in many cancer cell types, including breast cancer^6^, non-small cell lung cancer^7^ and ovarian cancer^8^. Typically, STAT3 is activated by a member of the Janus kinase (JAK) family, a non-receptor tyrosine kinase, resulting in nuclear translocation of phosphorylated STAT3 and transcriptional regulation of a diversity of genes^9^. In CCA cells, JAK2/STAT3 is activated in response to interleukin-6 (IL-6) and is involved in the regulation of cell proliferation and prevention of apoptosis through increased expression of anti-apoptotic genes such as the Bcl-2 family members^10^. Hence, targeting the JAK2/STAT3 signaling pathway may underpin novel methods for the CCA treatment.

Mesenchymal stem cells (MSCs) are multi-potent stem cells that could be isolated from several tissues and can differentiate into multiple lineages^11^. It was reported that MSCs also could regulate the secretion of several cytokines in immune response cells, and thus induce an anti-inflammatory and tolerant environment^12^. MSCs can be recruited to injured tissues and inflammatory areas, where they can maintain their multi-differentiation capacity^13^. In contrast to MSCs from other sources, chorion-derived MSCs (CH-MSCs) are viewed as a better alternative to MSCs for clinical applications owing to the painless collection procedure, high cell potency, low immunogenicity, the high paracrine potential for processes of tissue repair and strong tropism for tumors^14^. MSCs may stimulate a series of tumor signaling pathways by releasing a variety of cytokines that affect tumor cell development^15^; these signaling pathways may promote or suppress tumor proliferation, migration, invasion, or lead to cancer cell apoptosis^16^. All these characteristics make MSCs extremely attractive for targeted cancer therapy. Several studies have demonstrated the antitumor properties of MSCs in many types of cancer cell lines such as breast cancer^17^, gastric cancer^18^ and hepatocellular carcinoma^19^. However, the effect of MSCs on cholangiocarcinoma remains unclear, and the functional mechanisms of MSCs on CCA progression are poorly understood.

We therefore aimed to examine the effects of CH-CM on the proliferation and apoptosis of CCA cell lines in order to provide insight into the molecular mechanisms by which MSCs influence the biological functions of CCA cells. We hypothesized that CH-CM can suppress cell proliferation and promote apoptosis of CCA cells as well as inhibit JAK2/STAT3 signaling. These new findings may provide a basis for further clinical trials based on the development of new therapeutic methods and personalized approaches for treating CCA.

## Results

### Characterization of MSCs derived from chorion

Isolated CH-MSCs presented fibroblast-like morphology and the ability to adhere to plastic (Supplementary Fig. 1A). Flow cytometric analysis of cell surface antigens showed a high expression of the fibroblast markers CD73 (97.31 ± 1.44%), CD90 (93.44 ± 1.65%) and CD105 (93.37 ± 1.92%), and a lack of hematopoietic markers including CD34 (1.46 ± 0.69%) and CD45 (2.53 ± 1.05%) (Supplementary Fig. 1B). In addition, the cells were able to differentiate into multiple cell lineages, including adipocytes (Supplementary Fig. 1C) and osteocytes (Supplementary Fig. 1D).

### CH-CM suppresses the proliferation of CCA cells

**Figure 1:**
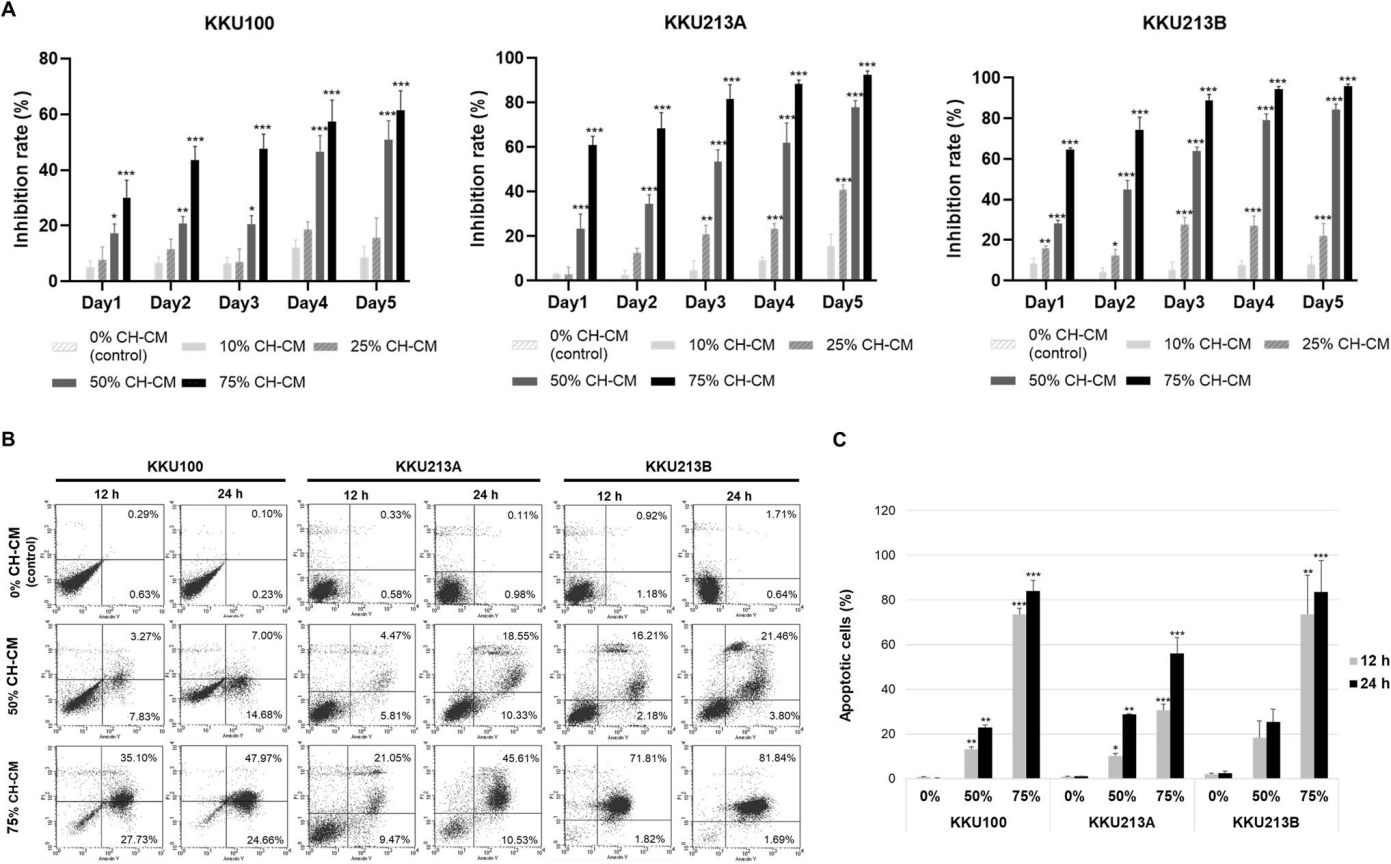
CH-CM increases the inhibition of the proliferation rate of CCA cell lines. (**A**) Three CCA cell lines were treated with CH-CM (0%, 10%, 25%, 50% and 75%). Every 24 h for 5 days, cell proliferation was measured by MTT assay. CH-CM significantly increased the proliferation inhibition rate of all three CCA cell lines. (**B**, **C**) Induction of apoptosis by CH-MSCs conditioned media in CCA cell lines**.** After culturing CCA cells with CH-CM (0%, 50%, 75%) for 12 h and 24 h, apoptotic cells were stained with annexin V-PI. Flow cytometric analysis showed that the number of apoptotic cells significantly increased in a dose-dependent manner compared with the control group. All data are presented as the mean ± SEM (n = 3) of a minimum of 3 donors. *p < 0.05, **p < 0.01, ***p < 0.001 compared to the control group.

To determine the effect of CH-CM on the proliferation of CCA cells, KKU100, KKU213A and KKU213B cells were treated with CH-CM at 0%, 10%, 25%, 50%, and 75% concentrations. MTT assay was used to assess proliferation every 24 h for 5 days. The data showed that the CH-CM significantly inhibited CCA cell proliferation. Specifically, the proliferation inhibition rate of KKU100 was significantly increased from 17.42 to 30.16%, the KKU213A cell line reached 23.31% and 60.94%, and KKU213B reached 28.29% and 64.55% after treatment for 24 h with 50% and 75% CH-CM, respectively (*p* < 0.001). On day 5, the inhibition rate was increased by 50.90% and 61.60% in KKU100, 77.81% and 92.47% in KKU213A as well as 84.42% and 96.02% in KKU213B after being culture cell with 50% and 75% CH-CM, respectively (*p* < 0.001). The data also indicated that CH-CM could suppress the proliferation of CCA cell lines in a dose- and time-dependent manner (Fig. 1A).

### CH-CM induces apoptosis of CCA cells

To investigate the effect of CH-CM on inducing apoptosis of CCA cells, the respective cell lines were cultured with CH-CM (0%, 50%, or 75%) for 12 and 24 h. After incubation, cells were stained with annexin V-FITC and propidium iodide solution and analyzed by flow cytometry. The results showed that CH-CM dramatically decreased surviving cells (from 96.03 to 20.84% for KKU100, from 90.01 to 38.01% for KKU213A, and 86.92 to 1.49% for KKU213B) and increased the apoptotic cells population (from 0.27 to 84.03% for KKU100, from 1.04% to 56.14% for KKU213A, and 2.35 to 83.53% for KKU213B) of CCA cell lines in a dose-dependent manner compared with the control group (0% CH-CM) (Fig. 1B,C).

### CH-CM induces loss of mitochondrial membrane potential (∆Ψm) in CCA cells

**Figure 2:**
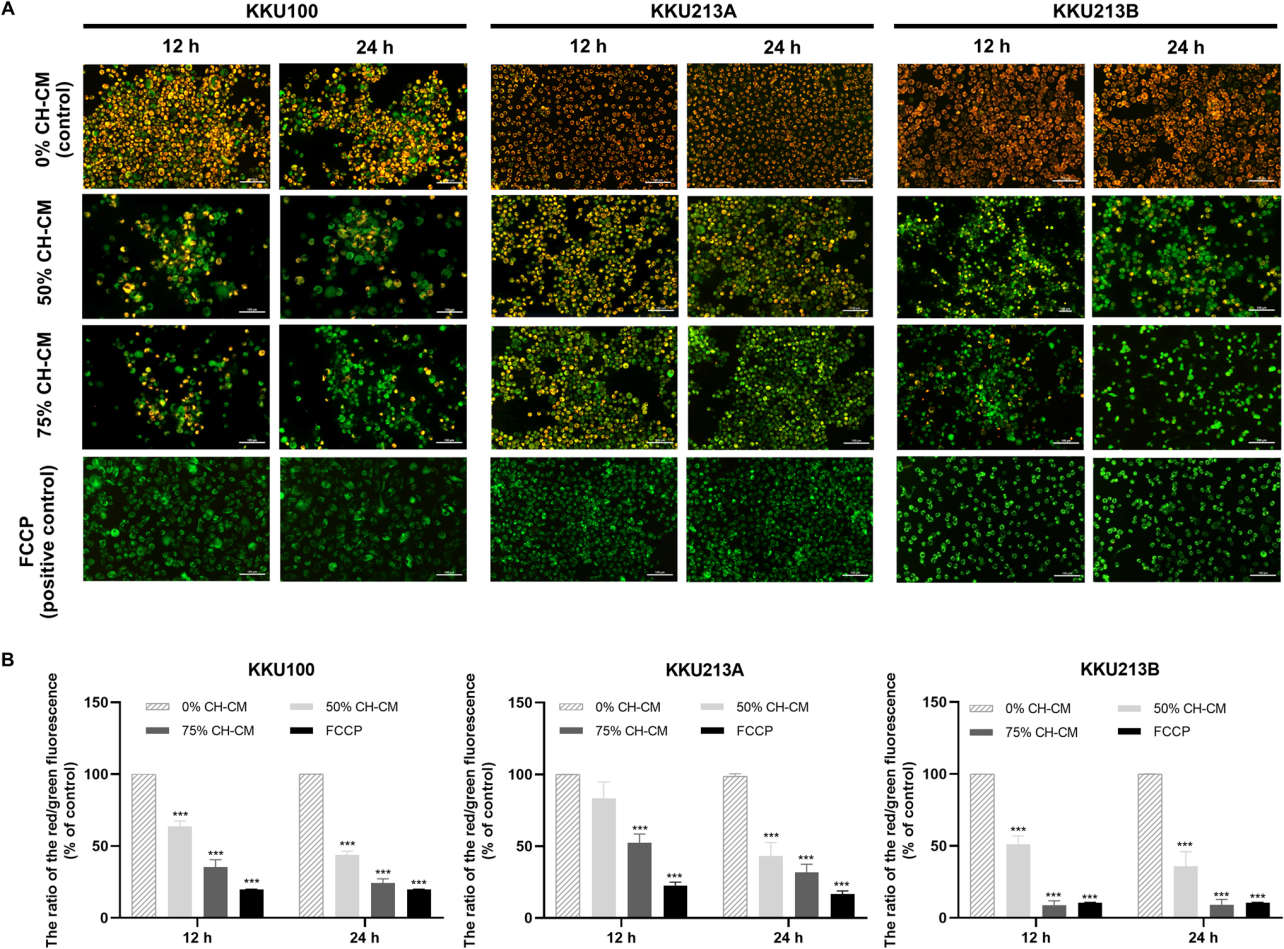
CH-CM induces the loss of mitochondrial membrane potential in CCA cell lines. CCA cells were treated with CH-CM at 0% 50% 75% for 12 h and 24 h and subsequently stained with JC-1 dye. FCCP was used as a positive control. (**A**) Fluorescence microscope analysis showed the increase of green fluorescence (unhealthy mitochondria) and reduction of red fluorescence (healthy mitochondria) in a dose- and time-dependent manner. (**B**) The quantitative analysis of JC-1 staining showed a significantly decreased red/green fluorescence ratio in all CCA cell lines after being cultured with CH-CM, thus indicating CH-CM-induced the loss of mitochondrial membrane potential in these cells. Data are expressed as mean ± SEM; n = 3. ***p < 0.001 compared to the control group.

The loss of mitochondrial membrane potential is a hallmark of apoptosis. The effect of CH-CM on the mitochondrial membrane potential in CCA cells was determined by JC-1 staining of CCA cells treated with CH-CM (0% 50% 75%) for 12 h and 24 h. Under these conditions, mitochondria showed an increase in monomeric or green fluorescence (representing unhealthy mitochondria) and a decrease in JC-1 aggregated or red fluorescence (healthy mitochondria) in a dose- and time-dependent manner (Fig. 2A). The quantitative analysis of JC-1 staining showed that all CCA cell lines presented a significantly decreased red/green fluorescence ratio when cultured with CH-CM (Fig. 2B). The data indicated that CH-CM promoted apoptosis of CCA cell lines by inducing loss of mitochondrial membrane potential.

### CH-CM promotes apoptosis in CCA cell lines by activating and regulating apoptosis-related proteins

**Figure 3:**
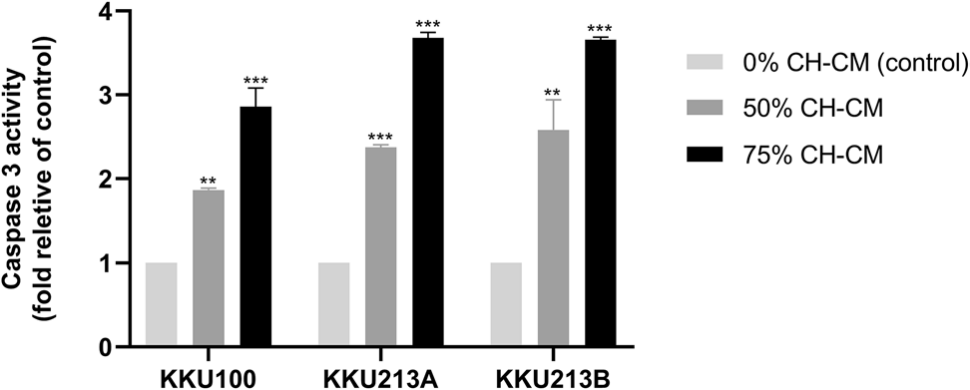
CH-CM induces activation of caspase-3 activity in CCA cell lines. The caspase-3 activity was determined by a microplate reader after culturing CCA cell lines with CH-CM (0%, 50%, 75%) for 8 h. The results showed that the caspase-3 activity in CCA cell lines was significantly higher after being cultured with CH-CM compared with control. Data are expressed as mean ± SEM; n = 3 **p < 0.01, ***p < 0.001 versus control.

**Figure 4:**
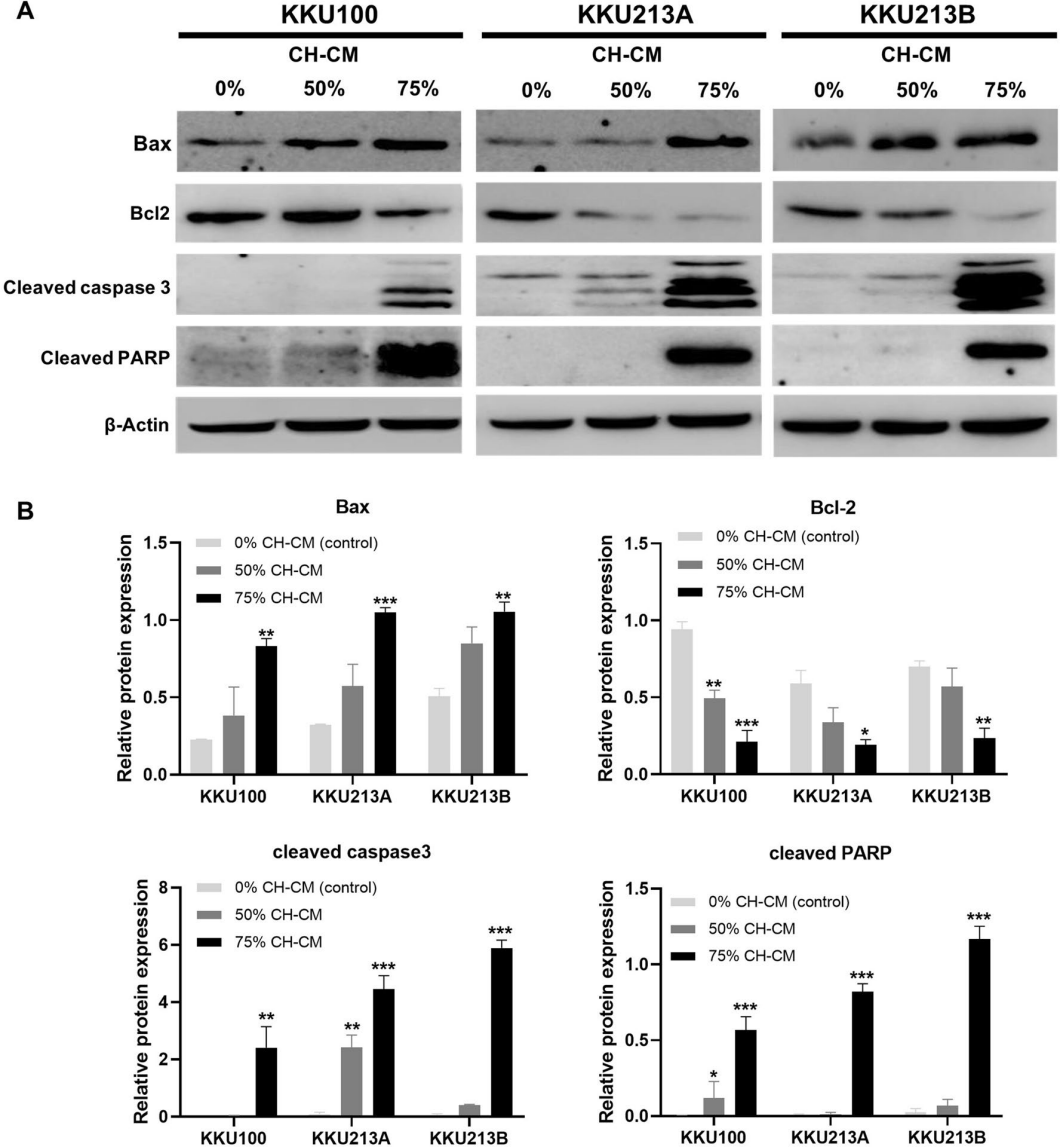
CH-CM regulates the expression of apoptosis-related proteins in CCA cell lines. (**A**) Increased expression of Bax, cleaved caspase-3 and cleaved PARP and decreased expression of Bcl-2 in CCA cells following culture in the presence of CH-CM at 50% and 75% compared with control (0%). (**B**) The relative protein level analysis showed significantly increased expression of Bax, cleaved caspase 3, and cleaved PARP and decreased expression of Bcl-2 in all CCA cell lines. All data are presented as the mean ± SEM. *p < 0.05, **p < 0.01, ***p < 0.001 compared to the control group. Full length western blots are provided in Supplementary Fig. 2.

**Figure 5:**
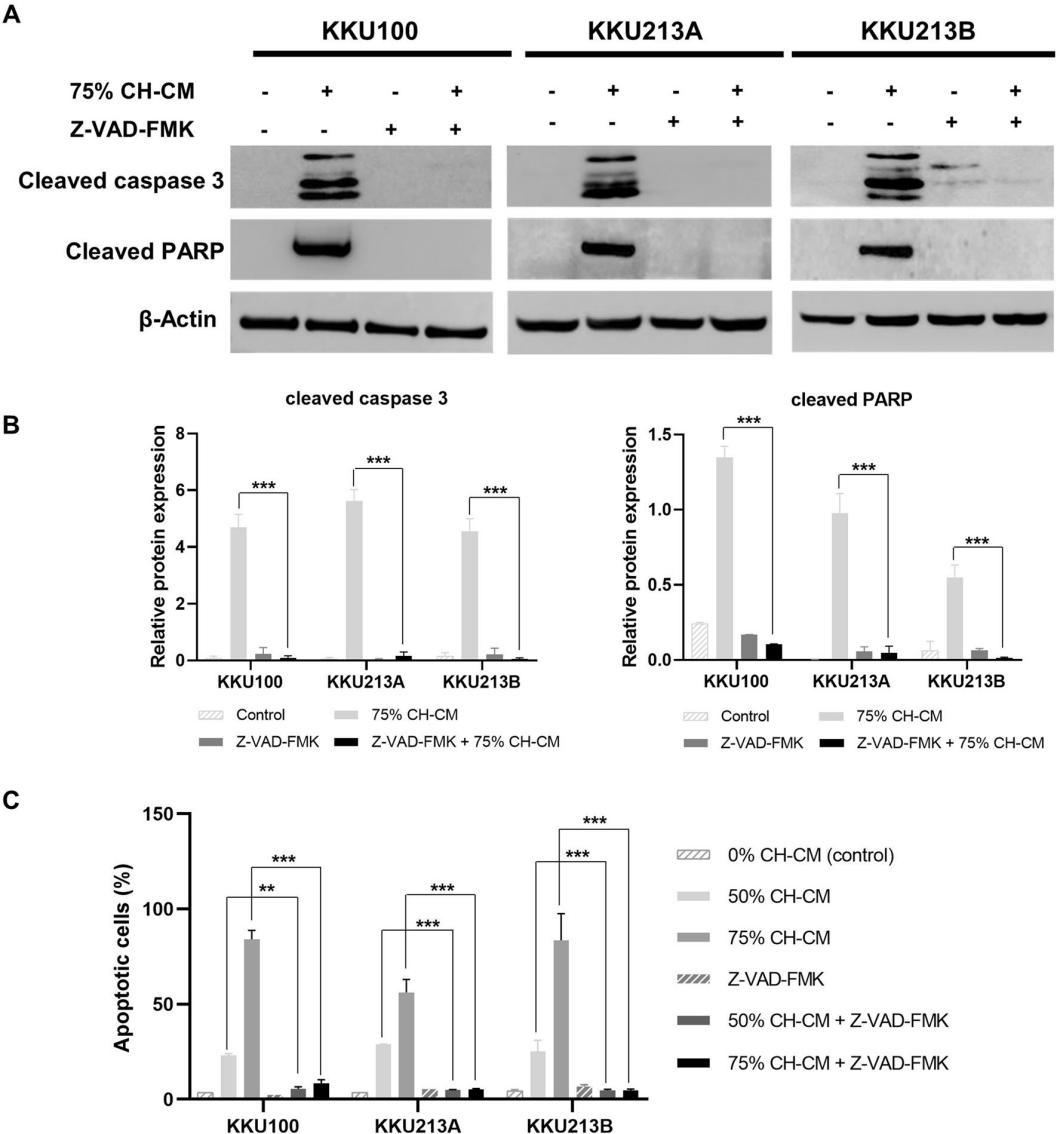
Pre-treatment with Z-VAD(OMe)-FMK prevents CH-CM-induced caspase-3 and PARP activation and cell apoptosis in CCA cell lines. CCA cell lines were pretreated with Z-VAD(OMe)-FMK (pan-caspase inhibitor) for 1 h and subsequently treated with CH-CM (0%, 50% and 75%) for 24 h. (**A**) The protein expression levels of cleaved caspase-3 and cleaved PARP were analyzed by western blot. (**B**) The relative protein level analysis showed that expression of cleaved caspase-3 and cleaved PARP were significantly reduced following pre-treatment of Z-VAD(OMe)-FMK (*p* < 0.001). (**C**) Flow cytometric analysis with annexin V–PI staining showed reduced CCA cell apoptosis following treatment with Z-VAD(OMe)-FMK. Full length western blots are provided in Supplementary Fig. 3. The representative dot plots of FACS analysis with annexin V–PI staining after treatment with Z-VAD(OMe)-FMK are shown in Supplementary Fig. 4.

To determine whether the MSC-induced apoptosis involves the caspase pathway, CCA cells were cultured with CH-CM (0%, 50%, 75%) for 8 h before assessing caspase-3 activation. The results showed that the caspase-3 activity in all CCA cell lines was significantly higher after culture with CH-CM than in control in a dose-dependent manner (Fig. 3). To investigate the molecular mechanism responsible for CH-MSC-induced apoptosis of CCA cell lines, the expression and level of apoptosis-related proteins Bax, Bcl2, cleaved caspase-3 and cleaved PARP were assessed by immunoblotting. Relative protein expression showed significantly increased expression of Bax, cleaved caspase-3 and cleaved PARP and decreased expression of Bcl-2 in CCA cell lines after being cultured with CH-CM at 50% and 75% compared with the control (Fig. 4A,B). Moreover, to confirm the effect of CH-CM on caspase-dependent apoptotic pathways, CCA cells were incubated in 75% CH-CM with and without pan-caspase inhibitor Z-VAD(OMe)-FMK. Pre-treatment with Z-VAD(OMe)-FMK prevented CH-MSCs induced caspase-3 and PARP activation (Fig. 5A,B) and cell apoptosis (Fig. 5C).

### CH-CM suppresses JAK2/STAT3 signaling in CCA cell lines and inhibits IL-6 induced JAK2/STAT3 activation

**Figure 6:**
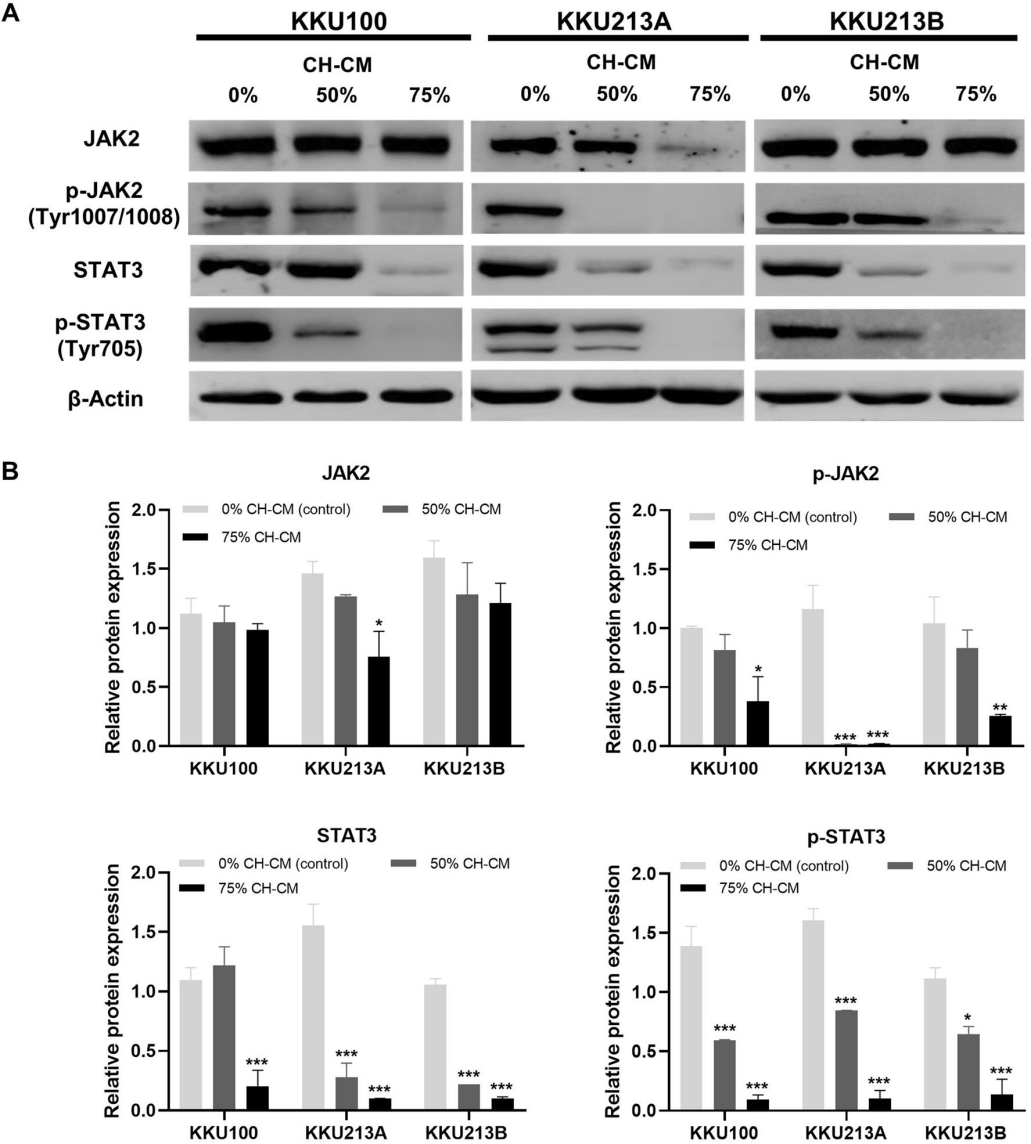
CH-CM suppresses JAK2/STAT3 signaling in CCA cell lines. CCA cell lines were cultured with CH-CM at 0%, 50%, and 75% for 8 h. (**A**) Western blot analysis showed that CH-CM decreases the expression of total and phosphorylated form of JAK2 and STAT3. (**B**) The relative expression analysis showed a dose-dependent manner and significant inhibition on the JAK2/STAT3 signaling in CCA cell lines (*p* < 0.001). Full length western blots are provided in Supplementary Fig. 5.

**Figure 7:**
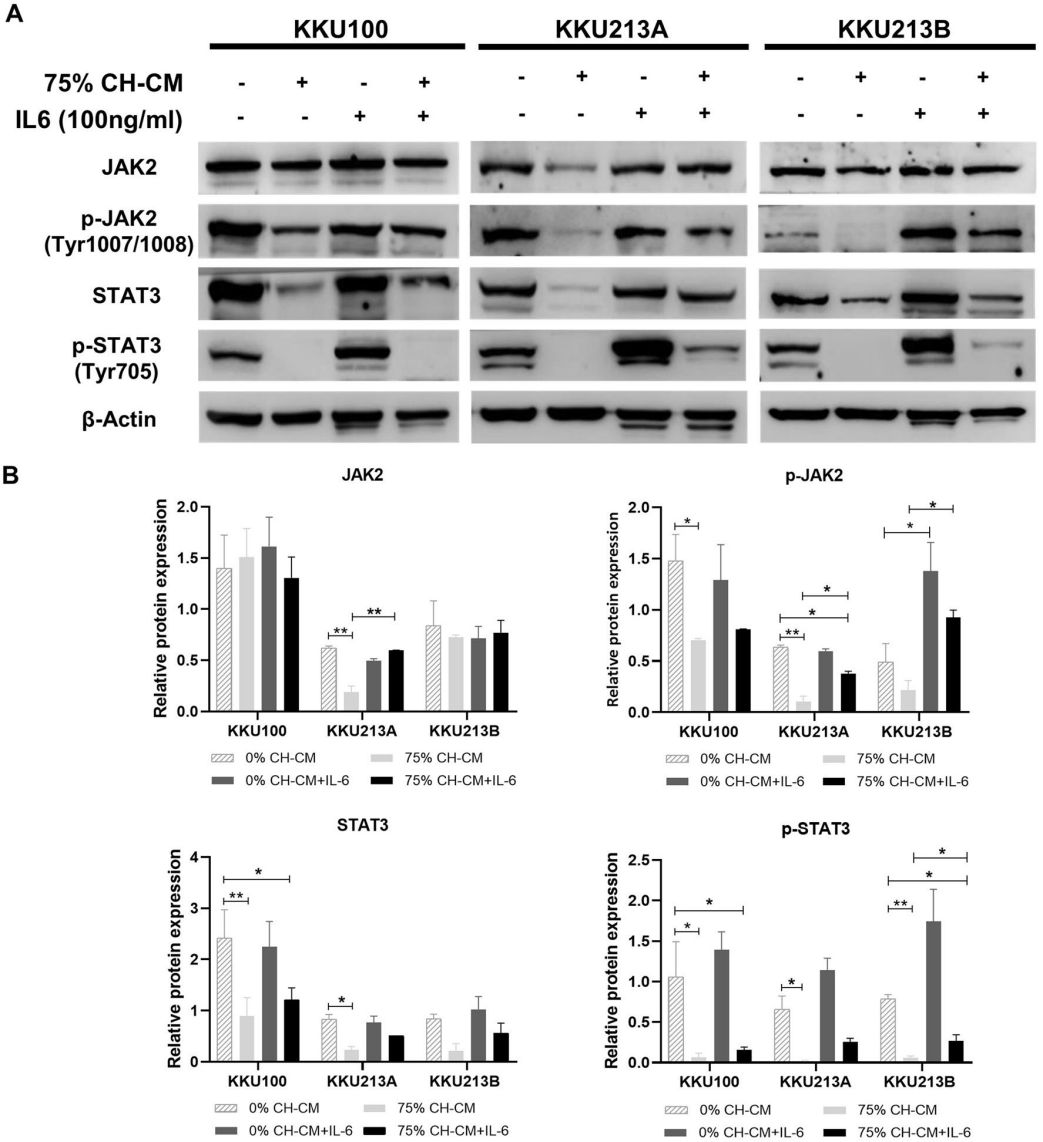
CH-CM inhibits IL-6-induced JAK2/STAT3 activation in CCA cell lines. CCA cells were cultured in the presence of 75% CH-CM for 8 h and then stimulated with IL-6 (100 ng/ml) for 30 min. (**A**, **B**) Western blot analysis showed that CH-CM inhibited IL-6-induced JAK2/STAT3 signaling in all CCA cell lines (*p* < 0.001). (**A**) Western blots showing the expression of phosphorylated JAK2 (active), total JAK2, phosphorylated STAT3 (active), total STAT3 and β-actin. (**B**) Relative protein levels of phosphorylated JAK2 (active), total JAK2, phosphorylated STAT3 (active) and total STAT3 (normalized to β-actin). Full length western blots are provided in Supplementary Fig. 6.

To investigate the mechanism of CH-CM induced CCA cell line apoptosis via JAK2/STAT3 signaling, the cells were cultured with CH-CM at 0%, 50%, and 75% for 8 h. CH-CM decreased the level of JAK2 (Y1007/Y1008) phosphorylation and Tyr705 phosphorylated STAT3 together with total JAK2 and STAT3, although total JAK2 in KKU100 and KKU213B were unaffected (Fig. 6A). The relative expression analysis showed a dose-dependent manner and significant inhibition of the JAK2/STAT3 signaling in CCA cell lines (Fig. 6B). Next, to examine whether CH-CM could inhibit IL-6-induced JAK2/STAT3 expression in CCA cells, CCA cells were pretreated with 75% CH-CM for 8 h and subsequently stimulated with IL-6 (100 ng/ml) for 30 min. Although the addition of IL-6 did not alter the levels of pJAK2 and pSTAT3 in all three CCA cell lines treated with 0% CH-CM, it significantly increased pJAK2 levels in both KKU213A and KKU213B treated with 75% CH-CM (Fig. 7). It is possible that the CCA cells treated with 0% CH-CM might express a higher level of IL-6 and pJAK2 than those treated with 75% CH-CM therefore, the addition of IL-6 only increased the pJAK2 level in CCA cells treated with 75% CH-CM but not in CCA cells treated with 0% CH-CM that already express a high level of IL-6 and pJAK2. In agreement with this assumption, the IL-6 also did not increase the pJAK2 levels in KKU100, which expressed much higher levels of pJAK2 compared with KKU213A and KKU213B, in the presence of either 0% or 75% CH-CM (Fig. 7). The IL-6 also increased the pSTAT3 levels in CCA cells treated with 75% CH-CM in a similar manner to those of pJAK2. However, the differences are not statistically significant due to the very low level of pSTAT3 in all CCA cells treated with 75% CH-CM (Fig. 7). A separate investigation revealed that the dosage of IL-6 used in this study only had a negligible effect on the cell viability of all CCA cell lines (Supplementary Fig. 7). Collectively, we believe that CH-CM suppressed the JAK2/STAT3 signaling in human CCA cells, and their suppressive effect might be mediated, at least in part, through the inhibition of IL-6.

## Discussion

Previous reports have evidenced that mesenchymal stem cells (MSCs) secrete a variety of cytokines from mesenchymal stem cells (MSCs) that inhibit the proliferation of many types of cancer cells, such as ovarian^20^, leukemia, and breast cancer cells^21^. However, the effect of MSCs on cholangiocarcinoma (CCA) remains unclear. Therefore, the present study has been conducted to investigate the effect of conditioned media from CH-MSCs on the regulation of proliferation and apoptosis mechanisms in three CCA cell lines (KKU100, KKU213A, KKU213B) isolated from primary tumors.

Firstly, we isolated MSCs from human chorion and confirmed that they had multi-lineage differentiation potential, including adipogenic and osteogenic differentiation; the cells had the usual features of MSCs as per criteria set by the International Society of Cellular Therapy, specifically the surface markers (positive for CD73, CD90, CD105 and negative for CD34 and CD45) and spindle-shaped morphology of the isolated CH-MSCs (fibroblast-like cells).

We then demonstrated that conditioned media from CH-MSCs could inhibit the proliferation of all three CCA cell lines. These results are consistent with findings previously reported^22^. Although some investigators have suggested that MSCs promote tumor progression and metastasis of CCA^23^, these discrepancies may be due to differences in the type of cell line, the heterogeneity of MSCs, the dose or timing of MSCs treated, or other unknown factors.

Further, to validate whether CH-CM could inhibit CCA cell lines proliferation by inducing apoptosis, annexin V/FITC-PI analysis was performed, and the results showed that CH-CM significantly increased the proportion of apoptotic cells in all CCA cell lines tested. Our results in this respect are in line with findings previously reported that human umbilical cord-derived MSCs and adipose-derived MSCs inhibit growth and promote apoptosis of hepatocellular carcinoma cells and gastric cancer cells, respectively^24^.

It is well known that cancer development results from an imbalance between cell growth and apoptosis manifested through disruption of the balance between pro-apoptotic (Bax) and anti-apoptotic proteins (Bcl-2), loss of the mitochondrial membrane integrity and reduction of the activity of caspases^25^. Furthermore, the present study revealed that CH-CM induced the loss of mitochondrial membrane potential through increased expression of Bax and decreased expression of Bcl-2 in CCA cells. CH-CM also induced activation of caspase-3 and increased the level of cleaved caspase-3 and PARP in CCA cell lines. The CH-CM-induced caspase-dependent apoptotic effect was confirmed using z-VAD-FMK, a universal inhibitor of caspases, which prevented MSCs-mediated apoptosis. Overall our findings indicate that CH-CM induced apoptosis in CCA cell lines is related to mitochondria-mediated caspase activity. These results are in line with recently reported findings that cytokines secreted from bone marrow-derived MSCs promote apoptosis through Bax and caspase cascade pathways in myelogenous leukemia cells^26^.

Our next aim was to investigate the mechanism of CH-CM by which the observed increase in apoptosis occurred in CCA cells. The JAK2/STAT3 signaling pathway plays a key role in mediating multiple effects of IL-6 on tumor cell proliferation, anti-apoptotic, invasion and metastasis^27^. The activation of STAT3 is involved in the regulation of transcription of several anti-apoptotic genes such as Bcl-2, Bcl-xl, and cyclin D1^28^. Conversely, increased levels of these anti-apoptotic proteins lead to cell survival, increased chemotherapy resistance, and suppressed apoptosis^29^. Our results showed that CH-CM reduced the phosphorylation of JAK2, an important tyrosine kinase that regulates STAT3 activation. Reduction of JAK2 phosphorylation resulted in inhibition of STAT3 activation. Moreover, CH-CM significantly inhibited IL-6-mediated JAK2/STAT3 activation in the CCA cells. JAK/STAT3 signaling is constitutively activated in cholangiocarcinoma and represents an important target for treating this disease^30^. Thus, while the inhibition of the JAK2/STAT3 signaling pathway has been proposed to offer therapeutic avenues for several types of cancer such as osteosarcoma^31^, gastric cancer^32^, ovarian cancers^33^, colon cancer^34^ as well as cholangiocarcinoma^35^, our findings suggest that JAK2/STAT3 signaling pathway may also be a good target for the therapeutic strategies applied to CCA.

Taken together, these results indicate, for the first time, that CH-MSCs release soluble factors that induce apoptosis of human cholangiocarcinoma cells. The CH-MSCs-derived factors also inhibit JAK2/STAT3 signaling, reduce the expression of BCL-2 anti-apoptotic protein, and increase BAX expression in those CCA cells. Change in the levels of those apoptotic genes correlated with the increased level of CCA cell apoptosis and might be responsible, at least in parts, for the negative effect of CH-MSCs on CCA cell viability.

In summary, our study revealed that conditioned media from human chorion-derived MSCs inhibit proliferation, suppress JAK2/STAT3 signaling, and promote apoptosis of CCA cells by inducing the mitochondrial apoptotic pathway. The data suggest that CH-CM has a potent anti-cancer effect on cholangiocarcinoma cells and thus provides opportunities for use in alternative cell therapy or in combination with a conventional chemotherapeutic drug for the effective treatment of CCA.

## Methods

### Cell lines and culture

Cells were provided from the Cholangiocarcinoma Research Institute, Faculty of Medicine, Khon Kaen University, Thailand. Three CCA cell lines, namely KKU100, KKU213A, and KKU213B established in the Faculty of Medicine, Khon Kaen University (Khon Kaen, Thailand), were used in all experiments. KKU100, a low-invasive cell line, was derived from extrahepatic CCA with poor differentiation^36^. KKU213B was a low-invasive cell line with well-differentiated squamous cell carcinomas, while KKU213A was a high-invasive cell line derived from intrahepatic CCA with poor differentiated^37^. KKU100 and KKU213B were cultured in Ham's F-12 nutrient mixture medium supplemented with 10% fetal bovine serum (FBS) and 1% antibiotic–antimycotic solution. KKU213A was cultured in Dulbecco's modified Eagle medium–high glucose (DMEM-H) supplemented with 10% FBS and 1% antibiotic–antimycotic solution in a humidified atmosphere of 5% CO_2_ at 37 °C.

### MSCs isolation and characterization

This study was approved by the Human Ethics Committee of Thammasat University No.1 (Faculty of Medicine; MTU-EC-DS-2-143-61) in accordance with the Declaration of Helsinki, the Belmont Report, and ICH-GCP. Informed consent was obtained from each patient. Also, all methods were carried out in accordance with relevant guidelines and regulations. Chorion tissues of healthy full-term pregnancies were obtained after labor with written informed consent from each participant. Chorion tissue was cut into small pieces and washed with 1X phosphate-buffered saline (PBS) to remove blood. Subsequently, the tissue was digested with 0.5% trypsin EDTA for 2 h at 37 °C with shaking. The cells and digested tissue were washed twice with 1X PBS and then cultured with DMEM supplemented with 10% FBS and 1% antibiotic–antimycotic solution in a humidified atmosphere of 5% CO_2_ at 37 °C. The phenotype of MSCs from the third passage was characterized by light microscopy, immunocytochemistry and differentiation capacity^38^. Specifically, to identify cell surface antigen markers, the absence of hematopoietic markers (anti CD34 and anti CD45) and the presence of MSCs markers (anti CD73, anti CD90, anti CD105) were checked by flow cytometry analysis. IgG was used as isotype control. The adipogenic and osteogenic differentiation potential of MSCs was examined by culturing MSCs in an adipogenic differentiation medium [Dulbecco’s Modified Eagle’s Medium (DMEM; GibcoBRL, USA) containing 10% fetal bovine serum (FBS; Invitrogen, USA), 2 mM l-glutamine (GibcoBRL, USA), 100 U/ml penicillin, 100 µg/ml streptomycin, 0.5 mM isobutylmethylxanthine (Sigma-Aldrich, USA), 1 µM dexamethasone (Sigma-Aldrich, USA), 10 µM insulin (Sigma-Aldrich, USA), 100 µM indomethacin (Sigma-Aldrich, USA)] and osteogenic differentiation medium [DMEM (GibcoBRL, USA) containing 10% FBS (Invitrogen, USA), 100 U/ml penicillin, 100 µg/ml streptomycin, 100 nM dexamethasone (Sigma-Aldrich, USA), 10 mM β-glycerophosphate (Sigma-Aldrich, USA), 50 µg/ml Ascorbic acid (Sigma-Aldrich, USA)], respectively. Oil red O (Sigma-Aldrich, USA) staining of adipogenic differentiated MSCs and Alizarin Red S (Sigma-Aldrich, USA) staining of osteogenic differentiated MSCs were performed.

### Preparation of conditioned medium

MSCs-derived from chorion (CH-MSCs) were cultured to 80% confluence, washed with 1X PBS twice, and incubated in FBS-free DMEM-H for 24 h. The CH-MSCs-conditioned media (CH-CM) was collected and centrifuged at 1000 g for 5 min to remove cell debris, then sterilized by filtering through a 0.22 µm filter. CH-CM was concentrated by lyophilizing for 72 h, reconstituted at 5X, and stored at − 80 °C until use.

### Proliferation assay

Cell proliferation was determined using an MTT assay^39^ (Sigma-Aldrich, USA). KKU100 (2.7 × 10^3^ cells/cm^2^), KKU213A (0.9 × 10^3^ cells/cm^2^) and KKU213B (0.9 × 10^3^ cells/cm^2^) cells were seeded and cultured overnight. CH-CM was added to the CCA cell culture medium to a final concentration of 0% (control), 10%, 25%, 50%, or 75%. Every 24 h for 5 days, MTT assays were performed according to the manufacturer's instructions. Briefly, 20 µl of 3 (4,5 dimethylthiazol 2 yl) 2,5 diphenyltetrazolium bromide (MTT) (5 mg/ml) was added to each well and incubated for 4 h. At the end of the incubation, all the solution was discarded, and 100 µl of dimethyl sulfoxide (DMSO) was added to solubilize the purple formazan crystals. Absorbance at 570 nm was measured by a microplate reader. The data are presented as the percentage of the proliferation inhibition rate, calculated with the following formula: Inhibition rate (%) = (1 − (OD_sample test_/OD_control_)) × 100%.

### Flow cytometry analysis of apoptosis

The apoptosis of CCA cell lines induced by CH-CM was analyzed using an annexin V/FITC kit (BioLegend, USA) according to the manufacturer's instructions. Apoptotic cells were analyzed by flow cytometry as described previously^40^. Briefly, CCA cell lines (1 × 10^5^ cells/cm^2^) were seeded and cultured overnight and then further cultured with CH-CM at various concentrations (0%, 50%, and 75%) for 12 h and 24 h. After incubation, cells and supernatant were harvested from each well. The cells were washed and resuspended in a binding buffer. After that, cells were double-stained with annexin V-FITC and propidium iodide solution and incubated in the dark for 15 min.

### Mitochondrial membrane potential assay

The JC-1 Mitochondrial Membrane Potential Assay Kit (Abcam, UK) was used to determine the mitochondrial membrane potential of CCA cells^41^. CCA cells (1 × 10^5^ cells/cm^2^) were cultured overnight and treated with CH-CM (0%, 50% or 75%) for 12 h and 24 h. Carbonyl cyanide 4-(trifluoromethoxy) phenylhydrazone (FCCP) was used as a positive control. The cells were harvested and stained with JC-1 dye. The JC-1 staining was assessed by fluorescence microscopy and measured for quantitative analysis by a fluorescence plate reader at Ex475 nm/Em530 nm (green fluorescence) and Ex475 nm/Em590 nm (red fluorescence). Mitochondrial depolarization was defined by the decrease of the red/green fluorescence ratio.

### Evaluation of caspase activation

The activity of caspase-3 was evaluated using a colorimetric assay kit (Abcam, UK)^42^. In brief, CCA cell lines were seeded (1 × 10^5^ cells/cm^2^) and cultured overnight, and subsequently treated with CH-CM (0%, 50%, or 75%) for 8 h. Cells were resuspended in 50 µl of chilled cell lysis buffer and cells were incubated on ice for 10 min, and then centrifuged at 10,000 × g for 1 min at 4 °C. The supernatants were collected, and their protein concentration was determined. The protein sample was adjusted to 200 µg per 50 µl cell lysis buffer for each sample and transferred into a 96-well plate. Reaction buffer (containing 10 mM DTT) and 50 µl of the 4 mM DEVD-p-NA substrate (200 µM final concentration) was added to each sample and incubated at 37 °C for 2 h. The activity of caspase-3 was measured for output (OD 405 nm) on a microplate reader.

The involvement of caspase activation in CH-CM-mediated apoptosis was confirmed by pretreatment of CCA cell lines with Z-VAD(OMe)-FMK (pan-caspase inhibitor) for 1 h prior to CH-CM treatment. Flow cytometric analysis was performed with annexin V–PI staining.

### Assessment of JAK2/STAT3 signaling

CCA cell lines (1 × 10^5^ cells/cm^2^) were seeded and cultured overnight, with the subsequent addition of CH-CM (0%, 50%, and 75%) for 8 h before cell collection. To examine whether CH-MSCs could inhibit IL-6-induced JAK2/STAT3 expression in CCA cells, CCA cells were pretreated with CH-CM (0% and 75%) for 8 h and then stimulated with IL-6 (100 ng/ml) for 30 min. The protein expression of STAT3, p-STAT3, JAK2 and p-JAK2 was analyzed by immunoblotting.

### Western blot analysis

CCA cell lines were lysed by RIPA lysis buffer (50 mM Tris, pH 7.4, 150 mM NaCl, 0.1% SDS, 1% TritonX-100, 1% sodium deoxycholate). Protein samples (20 µg/lane) were subjected to electrophoresis in 12% sodium dodecyl sulfate‑polyacrylamide gel (SDS-PAGE) and transferred onto nitrocellulose membranes. After blocking with 5% skimmed milk at room temperature for 1 h, blots were incubated with respective primary antibodies at 4 °C overnight and subsequently horseradish peroxidase (HRP)‑conjugated secondary antibody at room temperature for 1 h (1:10,000). Antibody complexes were visualized using an enhanced chemiluminescence detection system (ECL kit; 1705061) from Bio-Rad Laboratories. Primary antibodies were purchased from Cell Signaling Technology: Bcl-2-associated X protein; Bax (5023T), b-cell lymphoma protein 2; Bcl-2 (4223T), cleaved caspase-3 (9664T), cleaved poly (ADP-ribose) polymerase; PARP (5625T), signal transducer and activator of transcription 3; STAT3 (12640S), phospho-STAT3 (9145T), Janus kinase; JAK2 (3230T) and phospho-JAK2 (3776S). Beta-actin (β-actin) from Proteintech (catalog no. 66009-1-Ig) was used as a loading control.

### Statistical analysis

The statistical analyses were performed with GraphPad Prism Version 8.0.2 software (GraphPad Software, USA). The data were expressed as the mean ± standard error of the mean (SEM). Statistical significance comparisons between multiple groups were analyzed using one-way or two-way analysis of variance with the Tukey post hoc test. A *p* value < 0.05 was considered to indicate a statistically significant difference.

## Supplementary Information

Supplementary Information.

## Data Availability

All data generated or analysed during this study are included in this published article and its supplementary information files.
