# Supplementary material for: Human chorion-derived mesenchymal stem cells suppress JAK2/STAT3 signaling and induce apoptosis of cholangiocarcinoma cell lines
Source: Sci Rep. 2022 Jul 5;12:11341. doi: 10.1038/s41598-022-15298-0 (PMC9256624; doi:10.1038/s41598-022-15298-0)
Supplement: Supplementary file 1 — Supplementary Information. [file 41598_2022_15298_MOESM1_ESM.docx]

**Supplementary Information**

**Human chorion-derived mesenchymal stem cells suppress JAK2/STAT3 signaling and induce apoptosis of cholangiocarcinoma cell lines**

Tanachapa Jantalika^1,2^, Sirikul Manochantr^1,2^, Pakpoom Kheolamai^1,2^, Duangrat Tantikanlayaporn^1,2^, Weerachai Saijuntha^3^, Somchai Pinlaor^4^, Arthit Chairoungdua^5^, Luminita Paraoan^6*^ & Chairat Tantrawatpan^1,2*^

^1^Division of Cell Biology, Department of Preclinical Sciences, Faculty of Medicine, Thammasat University, Pathumthani 12120, Thailand; ^2^Center of Excellence in Stem Cell Research, Thammasat University, Pathumthani 12120, Thailand; ^3^Walai Rukhavej Botanical Research Institute (WRBRI), Biodiversity and Conservation Research Unit, Mahasarakham University, Maha Sarakham 44150, Thailand; ^4^Department of Parasitology, Faculty of Medicine, Khon Kaen University, Khon Kaen 40002, Thailand; ^5^Department of Physiology, Faculty of Science, Mahidol University, Bangkok 10400, Thailand; ^6^Department of Biosciences, Faculty of Arts and Sciences, Edge Hill University, BioSciences Building, St Helens Road, Ormskirk, L39 4QP, United Kingdom. Phone: +44 151 794 9038

^*^ authors for correspondence ([tchairat@tu.ac.th](mailto:tchairat@tu.ac.th); [Luminita.Paraoan@edgehill.ac.uk](mailto:Luminita.Paraoan@edgehill.ac.uk))


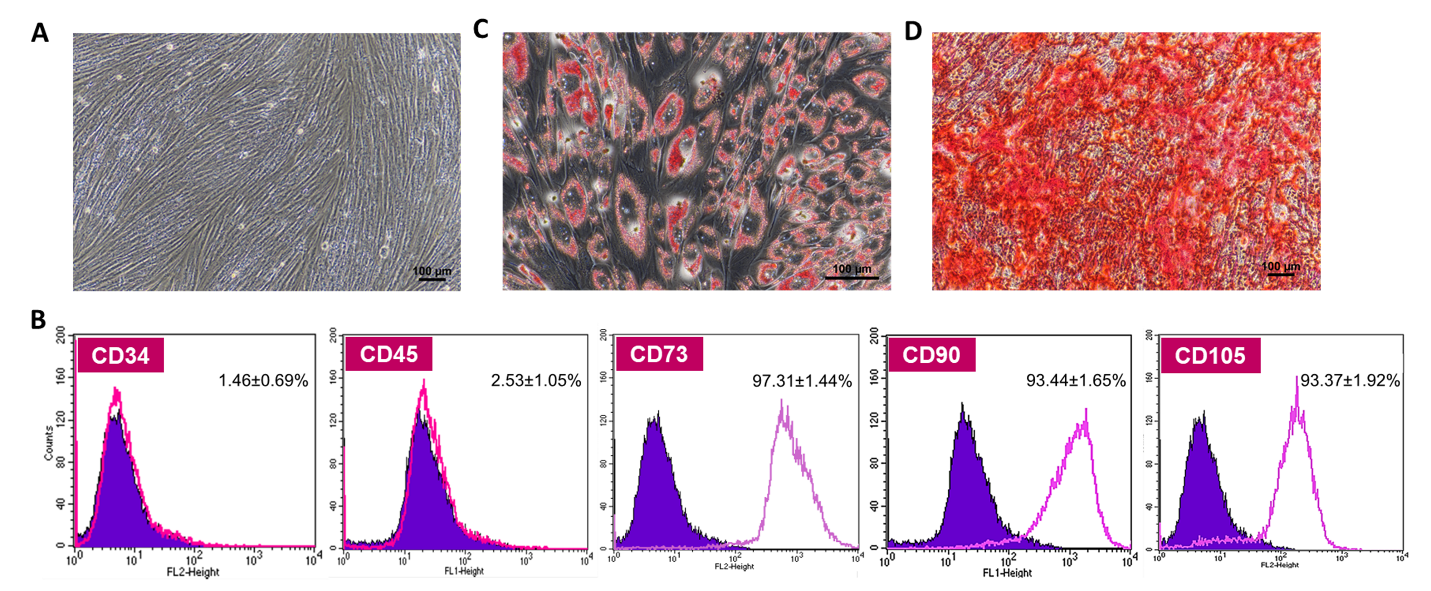


**Supplementary Figure 1.** The characterization of human chorion-derived mesenchymal stem cells (CM-MSCs). **A,** Isolated spindle-shaped CM-MSCs with fibroblast-like morphology. **B,** CH-MSCs were immunophenotypically characterized by flow cytometry being positive for CD73, CD90, CD105 and negative for CD34, CD45. IgG isotype was used as negative control (purple painted graph). **C,** Potential of CM-MSC for adipogenic differentiation demonstrated by Oil Red O staining (200× magnification). **D,** Potential of CM-MSC for osteogenic differentiation demonstrated by Alizarin Red S staining (100× magnification).

**BAX (~20 kDa)**


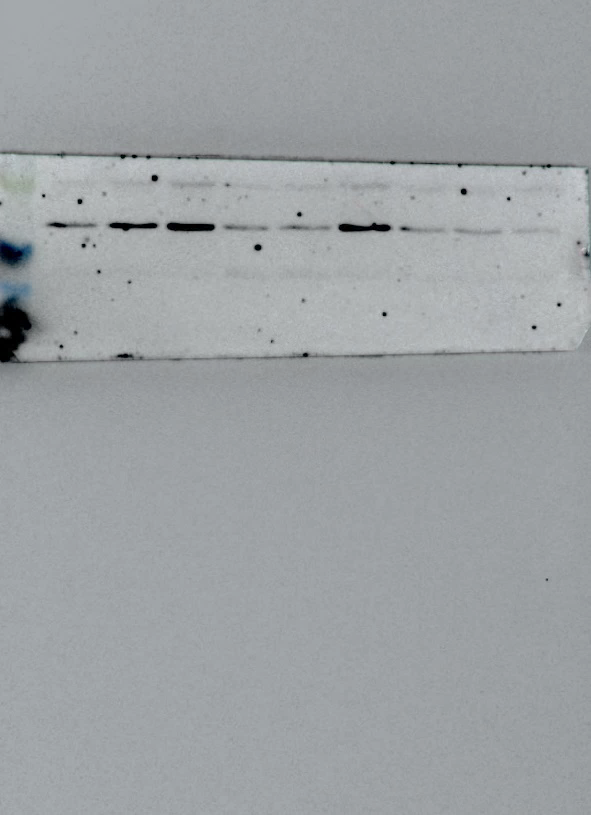

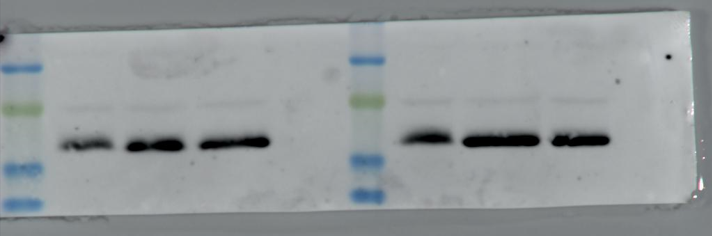


Bax

̴̴20 kDa

Bax

̴̴20 kDa

KKU213B

KKU213A

KKU100

**Bcl-2 (~26 kDa)**


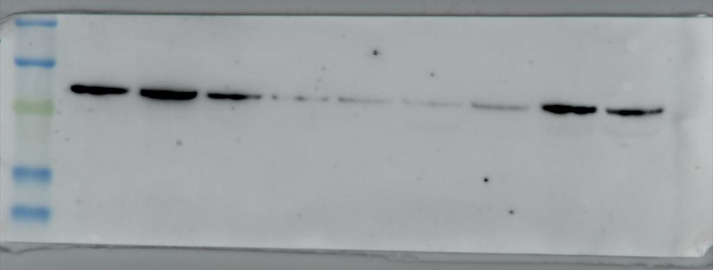

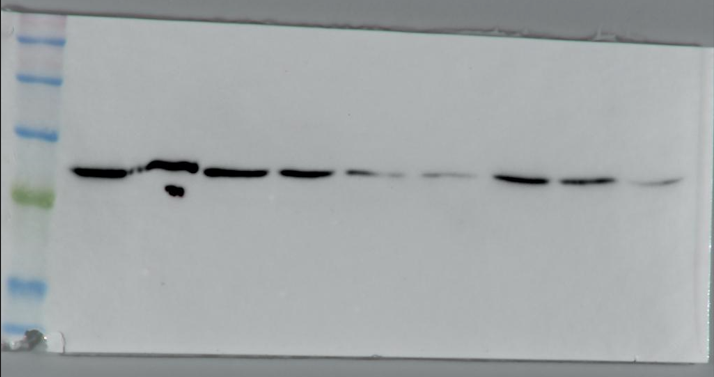


Bcl-2

̴̴26 kDa

Bcl-2

̴̴26 kDa

KKU100

KKU213A

KKU213B

**Cleaved-caspase 3 (~29/24/19/17 kDa)**


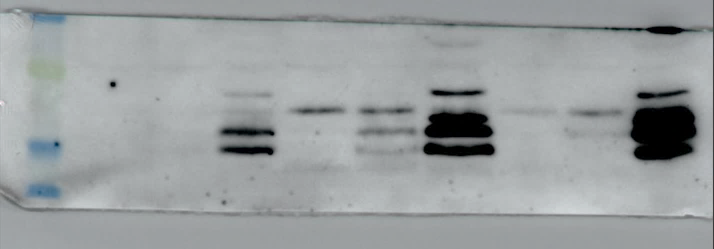


KKU213A

KKU213B

C-caspase 3

̴̴29/24/19/17 kDa

KKU100

**Cleaved PARP (~89kDa)**

KKU100


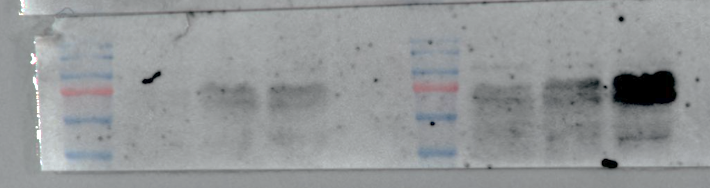

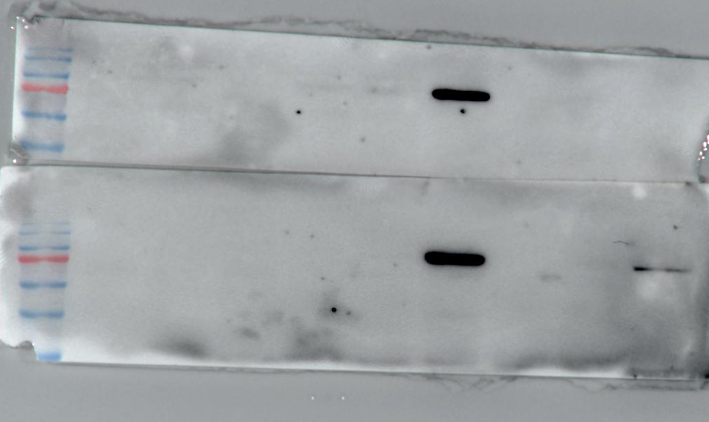


C-PARP

̴̴89 kDa

C-PARP

̴̴89 kDa

C-PARP

̴̴89 kDa

KKU213A

KKU213B

**β-actin (~42 kDa)**


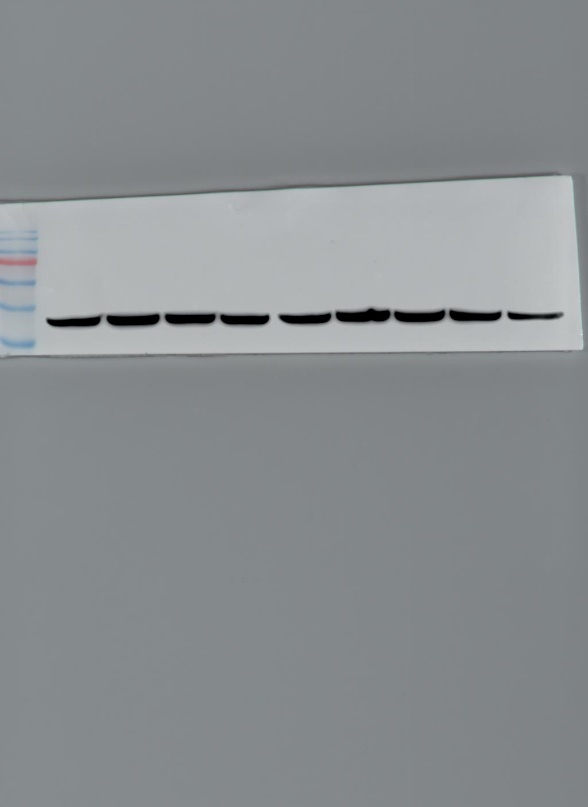

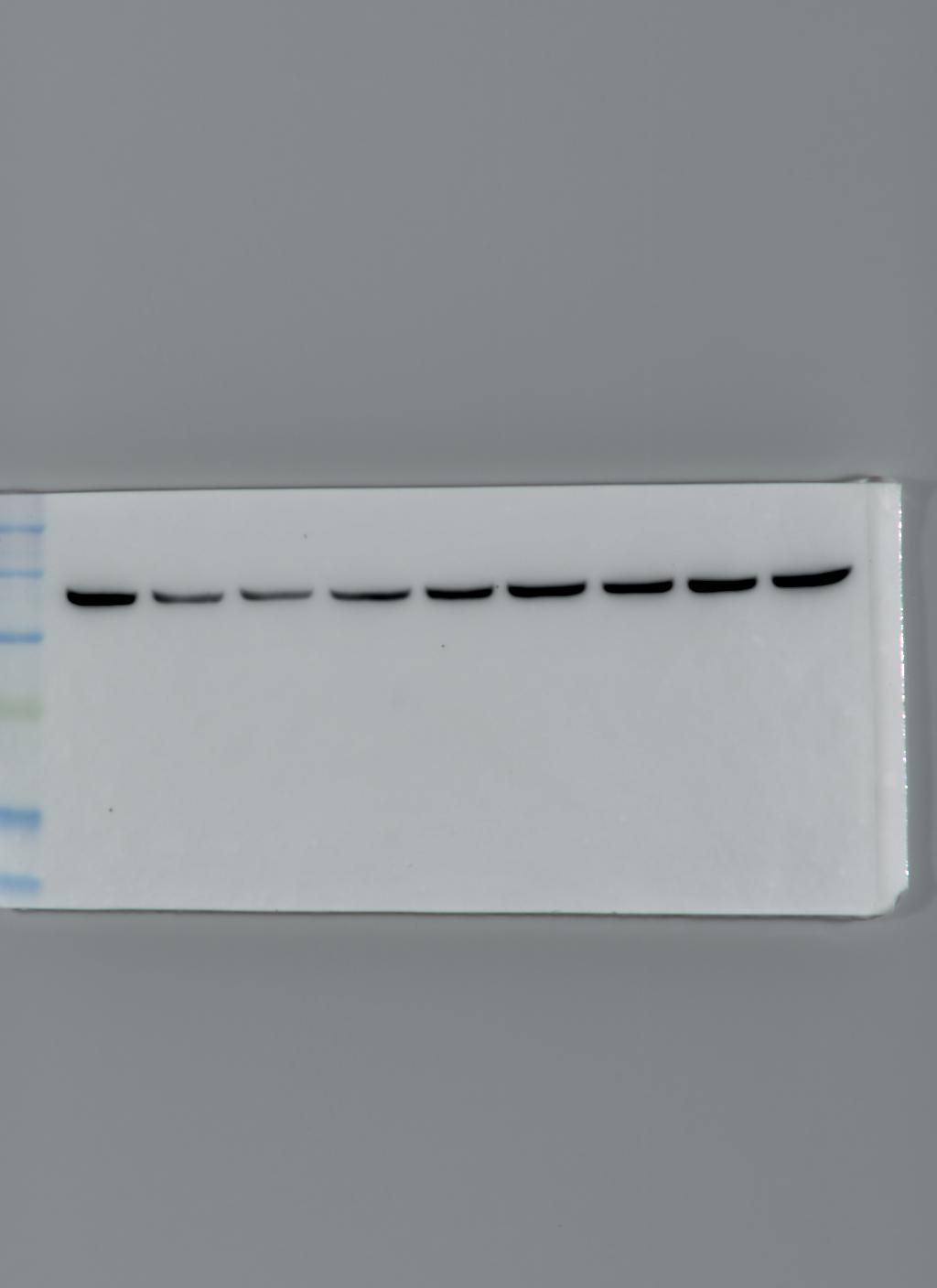


β-actin

̴̴42 kDa

KKU100

KKU213B

KKU213A

β-actin

̴̴42 kDa

**Supplementary Figure 2.** Full-length blots of western blot figures shown in Figure 4A

**Cleaved-caspase 3 (~29/24/19/17 kDa)**

KKU100


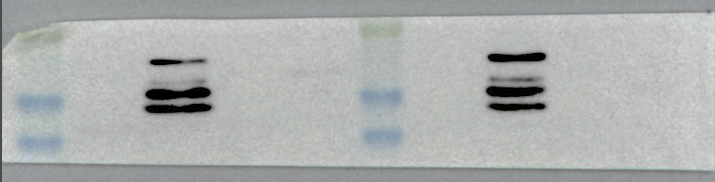


C-caspase 3

̴̴29/24/19/17 kDa


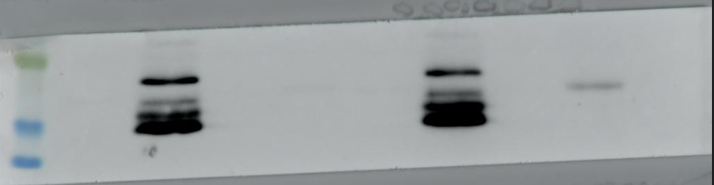


C-caspase 3

̴̴29/24/19/17 kDa

KKU213A

KKU213B


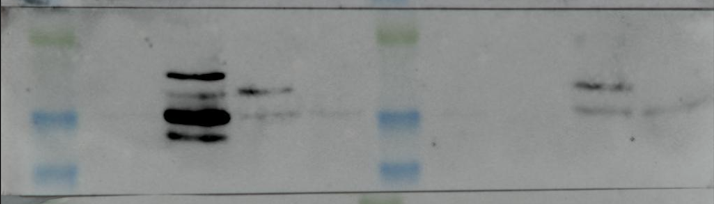


C-caspase 3

̴̴29/24/19/17 kDa

**Cleaved-PARP (~89 kDa)**

KKU100


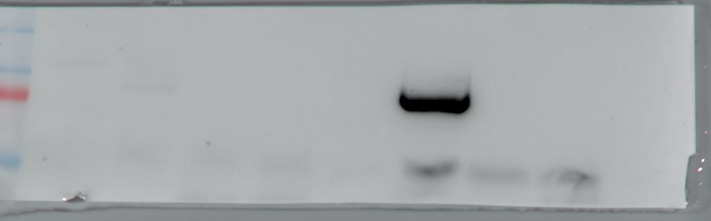


C-PARP

̴̴89 kDa


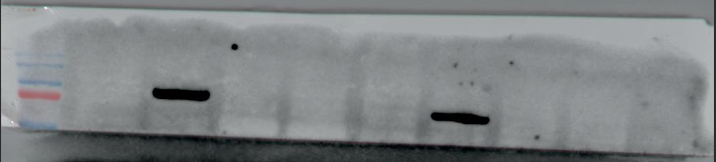


C-PARP

̴̴˜89 kDa

KKU213A


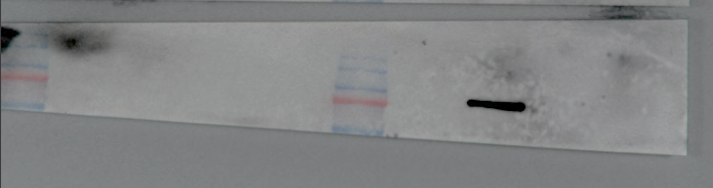


C-PARP

̴̴89 kDa

KKU213B

**β-actin (~42 kDa)**


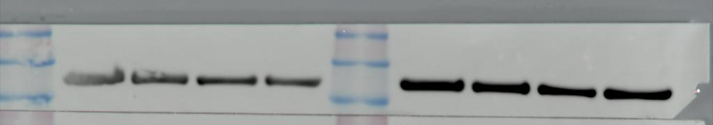


KKU100

Β-actin

̴̴42 kDa


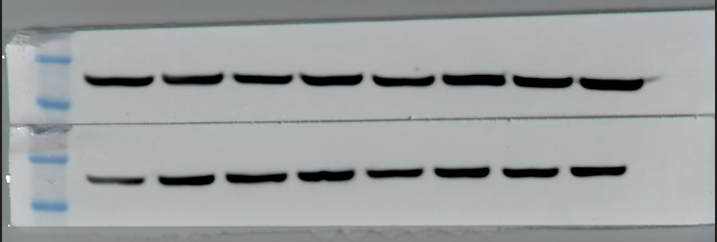


Β-actin

̴̴42 kDa

KKU213A


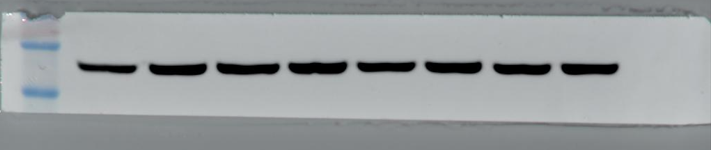


KKU213B

Β-actin

̴̴42 kDa

**Supplementary Figure 3.** Full-length blots of western blot figures shown in Figure 5A

**
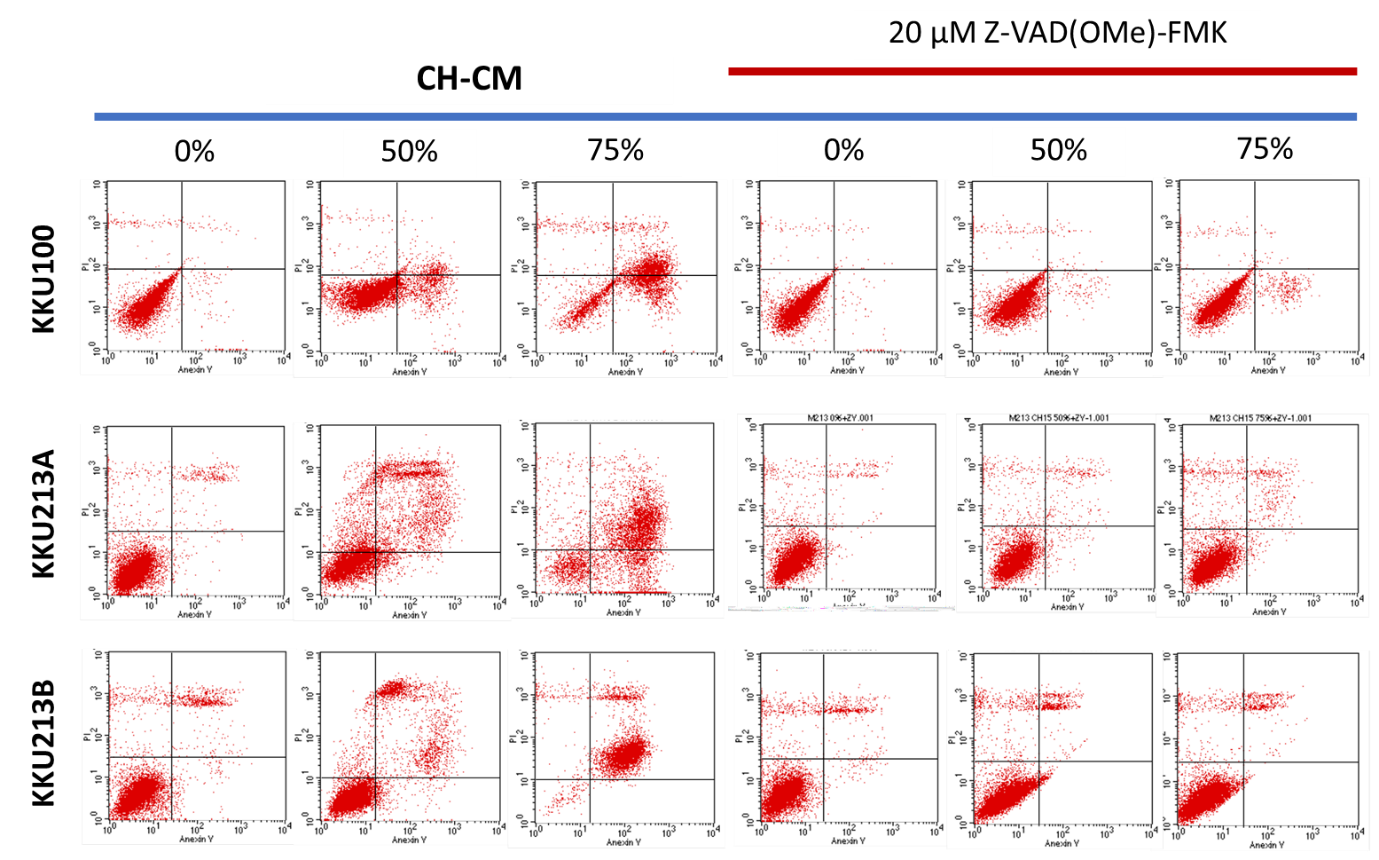
**

**Supplementary Figure 4.** The representative dot plots of FACS analysis with annexin V–PI staining showed reduced CCA cell apoptosis following treatment with Z-VAD(OMe)-FMK.

**JAK2 (~128 kDa)**

KKU100


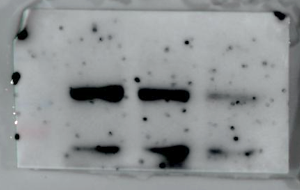

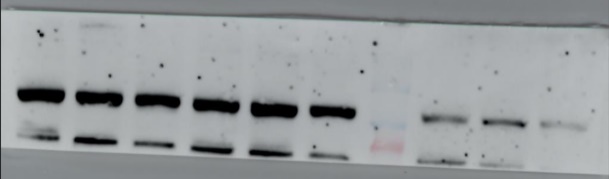

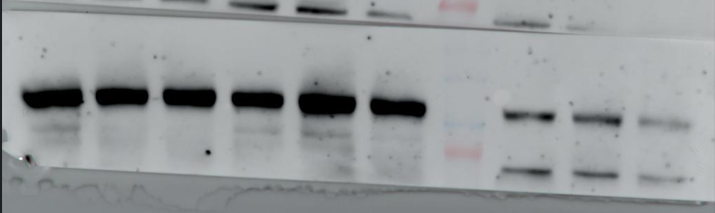


JAK2

̴̴128 kDa

JAK2

̴̴128 kDa

KKU213A

JAK2

̴̴128 kDa

KKU213B


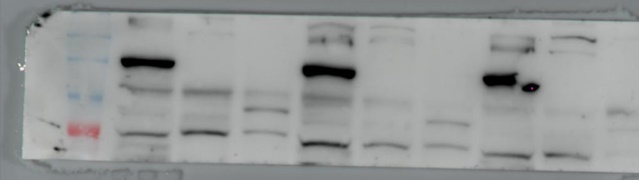

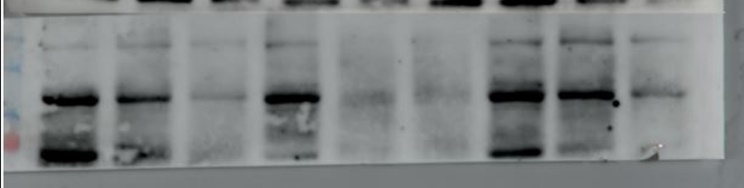
**p-JAK2 (~128 kDa)**

p-JAK2

̴̴128 kDa

KKU213A

p-JAK2

̴̴128 kDa

KKU100


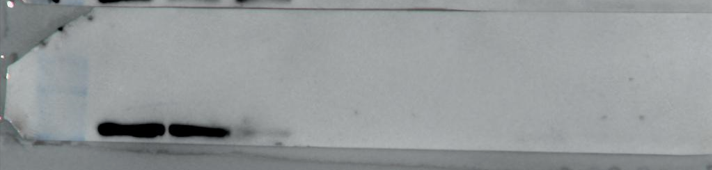


p-JAK2

̴̴128 kDa

KKU213B

**STAT3** **(~75-86 kDa)**


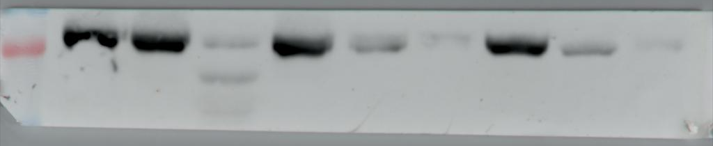


STAT3

̴75-86 kDa

KKU213B

KKU213A

KKU100


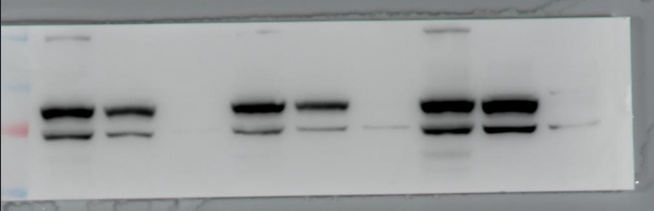

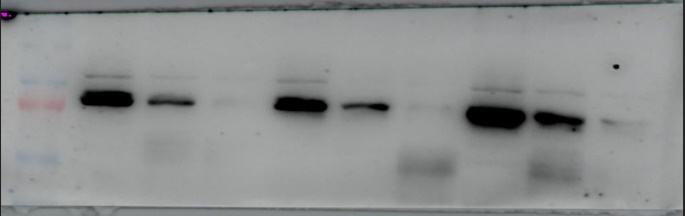
**p-STAT3 (~75-86 kDa)**

KKU213A

KKU100


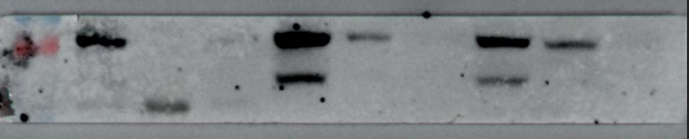


p-STAT3

̴75-86 kDa

KKU213B

**β-actin (~42 kDa)**


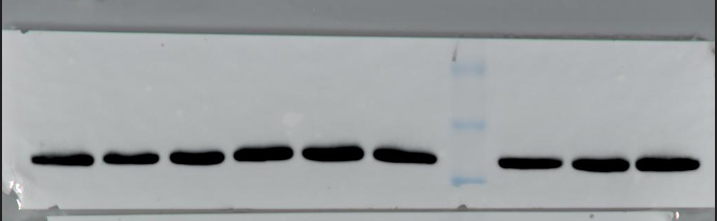

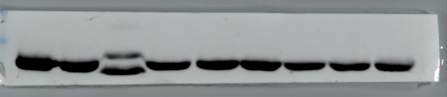


β-actin

̴42 kDa

KKU213B

KKU213A

KKU100

**Supplementary Figure 5.** Full-length blots of western blot figures shown in Figure 6A

**JAK2 (~128 kDa)**

KKU100

KKU213A


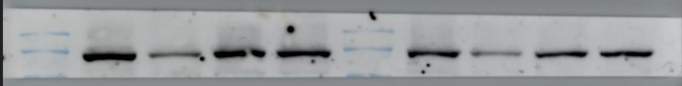

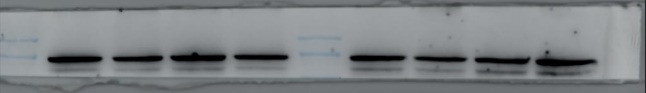


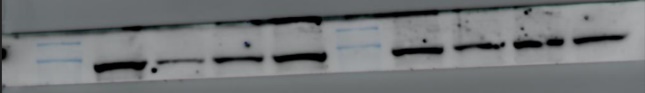


JAK2

̴̴128 kDa

KKU213B

**p-JAK2 (~128 kDa)**

KKU213A


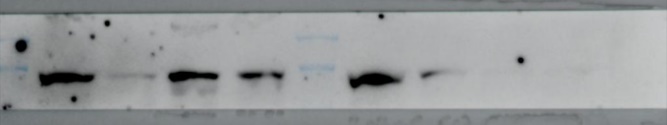

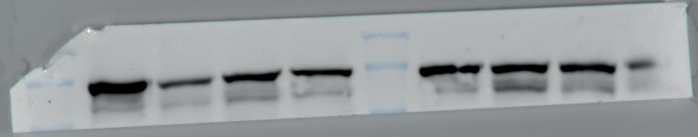


KKU100


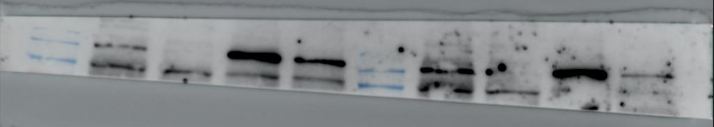


p-JAK2

̴̴128 kDa

KKU213B

**STAT3 (~75-86 kDa)**

KKU213A

KKU100


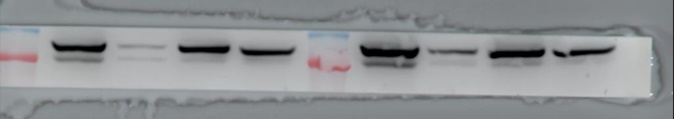

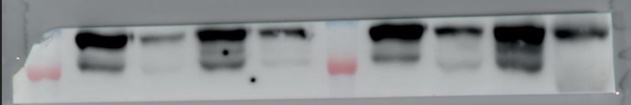


KKU213B


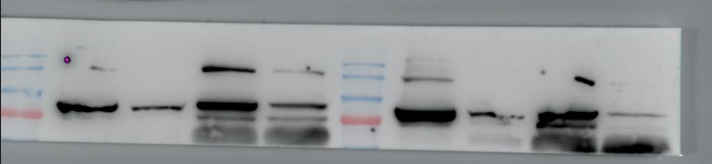


STAT3

̴75-86 kDa

**p-STAT3** **(~75-86 kDa)**

KKU213A


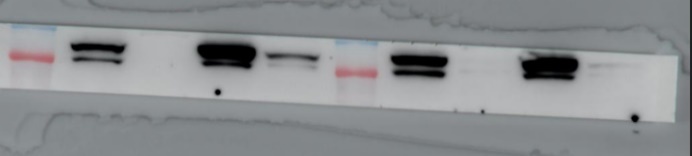

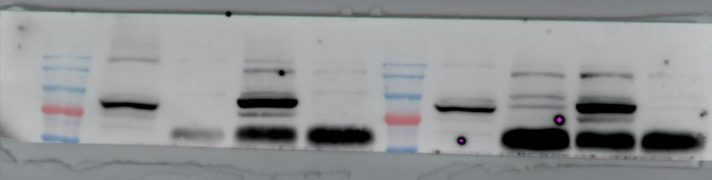


p-STAT3

̴75-86 kDa

KKU213B

KKU100


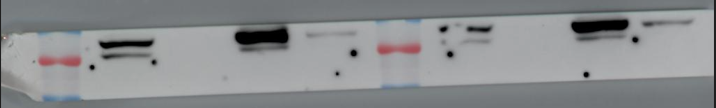


**β-actin (~42 kDa)**

KKU213A

KKU100


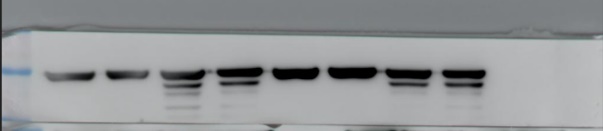

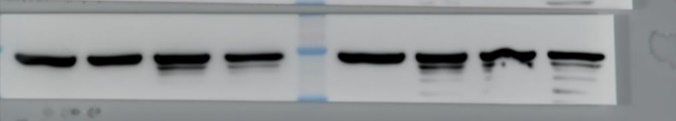


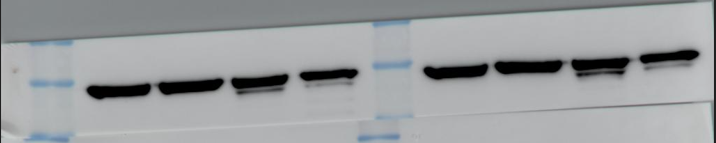


β-actin

̴42 kDa

KKU213B

**Supplementary Figure 6.** Full-length blots of western blot figures shown in Figure 7A

**
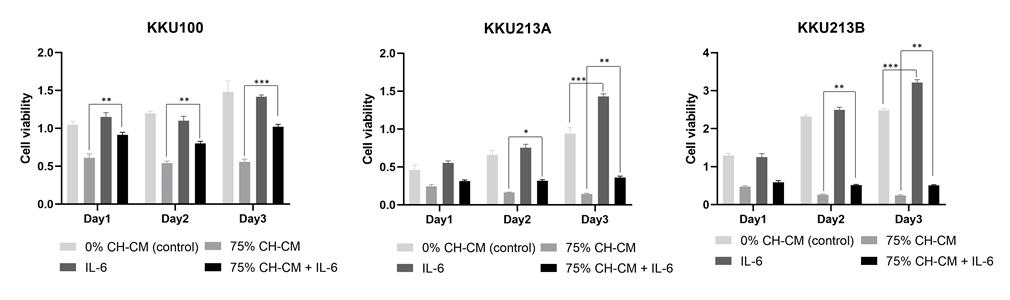
**

**Supplementary Figure 7.** Cell viability of CCA cell lines after treament with 0% CH-CM (as a control), 75% CH-CM, IL-6 alone, and 75% CH-CM + IL-6). MTT assay showed that the dose of IL-6 used in this experiment (100 ng/ml) only slightly affected the cell viability of CCA cells.
